# The Evaluation of Organizational Well-Being in An Italian Teaching Hospital Using the ANAC Questionnaire

**DOI:** 10.3390/ijerph16061056

**Published:** 2019-03-23

**Authors:** Claudio Giovanni Cortese, Federica Emanuel, Lara Colombo, Marco Bonaudo, Gianfranco Politano, Franco Ripa, Marilena Avanzato, Franca Dall’Occo, Antonella Rinaudo, Maria Michela Gianino

**Affiliations:** 1 Department of Psychology, University of Turin, 10124 Turin, Italy; claudio.cortese@unito.it (C.G.C.); lara.colombo@unito.it (L.C.); 2 Department of Philosophy and Educational Sciences, University of Turin, 10124 Turin, Italy; 3 Department of Public Health Sciences and Pediatrics, University of Turin, 10126 Turin, Italy; marco.bonaudo@unito.it (M.B.); mariola.gianino@unito.it (M.M.G.); 4 Department of Control and Computer Engineering, Politecnico of Turin, 10129 Turin, Italy; gianfranco.politano@polito.it; 5 Azienda Ospedaliero-Universitaria San Luigi Gonzaga, 10043 Orbassano, Italy; franco.ripa@regione.piemonte.it (F.R.); mavanzato@libero.it (M.A.); franca.dallocco@regione.piemonte.it (F.D.); a.rinaudo@sanluigi.piemonte.it (A.R.)

**Keywords:** organizational well-being, health care, teaching hospital

## Abstract

In Italy, the Italian National Anti-Corruption Authority (Autorità Nazionale Anti-corruzione—ANAC) has developed a questionnaire to assess the organizational well-being of employees within public agencies. The study aimed to explore the relationship among variables in the ANAC questionnaire: Several job resources (lack of discrimination, fairness, career and professional development, job autonomy, and organizational goals’ sharing) and outcomes of well-being at work, such as health and safety at work and sense of belonging. The research was carried out among workers in an Italian hospital in Northwest Italy (*N* = 1170), through an online self-report questionnaire. Data were grouped into two job categories: Clinical staff (*N* = 939) and non-clinical staff (*N* = 231). The hypothesized model was tested across the two groups through multi-group structural equation modeling. Results showed that health and safety at work and sense of belonging had significant positive relationships with the other variables; some differences emerged between the determinants of the two outcomes and among groups. The study aims to identify some reflections and suggestions regarding the assessment of well-being in the health care sector; implications for practice are identified to promote organizational well-being and health in organizations.

## 1. Introduction

Several studies highlighted the negative effects of work-related stress on workers’ health and well-being and on organizations’ productivity and dynamism [1,2,3]; moreover, research has shown the importance of job resources, for example, social support, to sustain employees’ well-being and satisfaction [4,5,6]. In fact, satisfied and engaged employees tend to be more productive, creative, and enthusiastic and this has a direct and positive impact on the functioning of the organization and strongly affects organizational performance [7,8].

The aim of this study was to analyze the relationship between some job resources and outcomes of well-being at work in an Italian teaching hospital, according to Italian legislation and indications.

### The Evaluation of Organizational Well-Being according to the ANAC Recommendations

In Italy, according to European indications, legislation has emphasized the importance of monitoring organizational well-being and work-related stress, ensuring health and quality of life at the workplace. Legislative Decree 81/2008 requires all Italian public and private organizations to assess and manage work-related stress risk. The evaluation of work-related stress allows organizations to improve and to evaluate well-being and quality of life at work too, as shown in several studies on this topic [9,10,11]. Moreover, Legislative Decree 150/2009 requires public agencies to conduct surveys involving all workers in order to detect: The organizational well-being, defined as “the state of health of an organization in relation to the quality of life, the degree of physical, psychological and social well-being of the working community, aimed at the qualitative and quantitative improvement of its results” [12] (pp. 3–4); the degree of sharing of the performance evaluation system approved by the organization and implemented within it; and the evaluation of the direct supervisor. According to the Autorità Nazionale Anti-Corruzione (ANAC; National anti-corruption authority and for the evaluation and transparency of public agencies) recommendations [12], these surveys provide organizations with information and data useful to implement solutions and enhance performance. ANAC has proposed to the public agencies a questionnaire to use for this purpose; in this study, we used some data from this tool to reflect on well-being at work. Among the theoretical models able to understand the numerous aspects affecting well-being at work, the job demands-resources theory (JD-R theory) [6] has received much attention by scholars. The model assumes that well-being is influenced by two main categories of factors, job demands and job resources: Job demands are mainly responsible for health degradation processes, while job resources are mainly responsible for motivational processes. The flexibility of the model permits the theory to be applied to all work environments and occupations, identifying specific job demands and job resources. Job demands are “physical, psychological, social, or organizational aspects of the job that require sustained physical and/or psychological (cognitive and emotional) effort or skills and are therefore associated with certain physiological and/or psychological costs” [13] (p. 312). Job demands are not negative by definition; they become job stressors when meeting those demands requires a high that the person cannot adequately cope with. Job resources represent the second set of job characteristics and “refer to those physical, psychological, social, or organizational aspects of the job that are either/or: Functional in achieving work goals; reduce job demands and the associated physiological and psychological costs; stimulate personal growth, learning, and development” [13] (p. 312). High levels of job-related stressors and a lack of job resources may negatively affect employees’ well-being [6,13].

In this study, we explored the relationship between some job resources and outcomes of well-being at work, specifically health and safety at work and sense of belonging, measured by different areas of the ANAC questionnaire. Health and safety at work is a key priority for occupational health psychology and for organizations too; it refers to the actions that an organization puts in place to promote workers’ well-being and health, and to sustain employees in everyday activities [14,15]. High levels of health and safety at work are also observable in a safe and comfortable work environment, in the management’s attention and responsiveness to workers’ needs, and in specific training on safety. This study considered another outcome, the sense of belonging, which indicates the level of membership and the feeling of cohesion that employees feel about their organization [16,17]. Employees’ organizational sense of belonging is also defined as workers’ identification with the organization’s goals and values; several studies reported positive behavioral consequences at the organizational level when sense of belonging was strong, such as high-quality health care and engagement [18,19,20].

As previously pointed out, this study explores the relationship among some variables, precisely job resources, according to the JD-R theory; job demands in fact were not considered in the ANAC tool. The job resources included in the study (lack of discrimination, fairness, career and professional development, job autonomy, and organizational goals’ sharing) are briefly presented below.

Lack of discrimination refers to an absence of discrimination in the organization, with respect to, for example, gender, religion, or political affiliation; perceived discriminations in workplace can be detrimental to health and lead to increased psycho-physical symptoms of stress and/or discomfort at work [21,22].

Fairness pertains to an objective and equitable treatment in the organization, for example, in relation to the allocation and distribution of resources, and to the decision-making processes or information sharing [23]. This job resources is related to citizenship behavior [24] and can make significant contributions to employees’ well-being [25].

Career and professional development refers to how much the organization makes accessible criteria and opportunities for growth within it. Several studies show that when career opportunities and criteria are clear, employees present higher levels of well-being and lower levels of work-related stress [26,27].

Job autonomy [28] concerns the degree to which a job provides workers with freedom, independence, and self-government in scheduling activities and determining procedures used in carrying it out, as well as the degree of control over the activities’ sequence and over the criteria used to evaluate performance [29,30].

Organizational goals’ sharing relates to how the organization communicates and transfers its goals, mission, and values. Chan and Reich [31] state that management uses organizational goals to enhance the company and motivate its members. Research suggests that sharing organizational goals can promote membership and clear communication within the organization.

**Figure 1:**
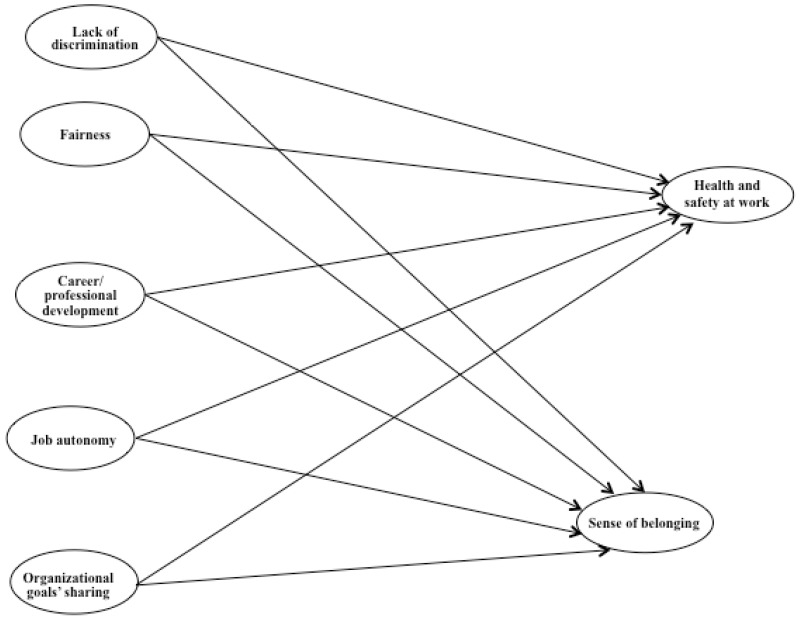
Hypothesized model.

The aim of the study, as pointed out previously, was to investigate the relationship between job resources (lack of discrimination, fairness, career and professional development, job autonomy, and organizational goals’ sharing) and health and safety at work and a sense of belonging among the employees at an Italian teaching hospital, using data by the ANAC questionnaire. Figure 1 shows the hypothesized model.

According to the JD-R theory [6], job resources are mainly responsible for motivational processes and well-being dynamics; thus, we expected that:

**Hypothesis 1:**  Job resources (lack of discrimination, fairness, career and professional development, job autonomy, and organizational goals’ sharing) have a positive relationship with health and safety at work.

**Hypothesis 2:**  Job resources (lack of discrimination, fairness, career and professional development, job autonomy, and organizational goals’ sharing) have a positive relationship with sense of belonging.

Moreover, we attempted to identify possible differences between job categories, because different types of tasks can generate diverse emotional and psychological dynamics, and thus different management challenges [32]. Participants were divided into clinical staff and non-clinical staff subsamples. The perspective from which we studied these possible differences is explorative; therefore, we did not define specific hypotheses regarding this.

## 2. Materials and Methods

### 2.1. Participants and Procedure

The study was carried out among workers in an Italian hospital in Northwest Italy. It is the second teaching hospital at the University of Turin’s School of Medicine, with more than 400 beds and approximately 1400 workers. 

The study was conducted in accordance with the Helsinki Declaration [33] and Italy’s data protection regulation. The research project was shared with the trade unions and approved by the Company Board of Directors. Because there was no medical treatment or other procedures that could cause psychological or social discomfort to participants, no additional ethical approval was required. Participation was voluntary and without remuneration. Anonymity and confidentiality in collecting, analyzing, and publishing the data were guaranteed. The aim of the study was explained by sending an e-mail from management and a communication published in the intranet magazine.

1170 employees (87% of all workers) fulfilled the online self-report questionnaire. Data were grouped into two job categories: (1) Clinical staff, including medical doctors; technical executives (i.e., pharmacists, dieticians, and chemists); nurses and allied health professionals (i.e., radiographers, therapists, and laboratory technicians) and (2) non-clinical staff, including other executives’ officer and administrative staff (i.e., engineers, lawyers, analysts, statistical, and administrative staff).

The clinical staff sample (*N* = 939, 80.3%) included 75% females and 25% males. The age distribution was: 3.1% up to 30 years, 17.4% from 31 to 40 years, 42.0% from 41 to 50 years, 31.8% from 51 to 60 years, and 5.7% over 60 years. The organizational tenure was: 4.2% up to 5 years, 16.1% from 5 to 10 years, 34.1% from 11 to 20 years, and 45.6% over 20 years. 23.1% of the participants were directors/supervisors.

The non-clinical staff sample (*N* = 231, 19.7%) included 61.4% females and 38.6% males. The age distribution was 0.9% up to 30 years, 9.3% from 31 to 40 years, 31.6% from 41 to 50 years, 51.1% from 51 to 60 years, and 7.1% over 60 years. The organizational tenure was 1.3% up to 5 years, 11.6% from 5 to 10 years, 24.9% from 11 to 20 years, and 62.2% over 20 years. 4.5% of the participants were directors/supervisors.

### 2.2. Measures

The ANAC questionnaire [12] was composed by different areas of investigation: (A) Health and safety at work and work-related stress, (B) discrimination, (C) fairness in my administration, (D) career and professional development, (E) my work, (F) my colleagues/the person in charge of my business, (G) the context of my work, (H) the sense of belonging, (I) the image of my administration, (L) my organization (evaluation system), (M) my performance (evaluation system), (N) system operation (evaluation system), (O) my boss and my growth (direct supervisor), and (P) my boss and equity (direct supervisor). In this study, we considered A, B, C, D, E, H, and L areas; all items were scored on a 6-point Likert scale (1 = strongly disagree, 6 = strongly agree).

ANAC did not provide indications or measurement for psychometric and reliability properties of the toll. For this reason, in addition to the structural equation model, we conducted confirmatory factor analysis for each scale. Table 1 reports the Confirmatory Factor Analysis (CFA) results and Cronbach’s alpha in this study.

Health and safety at work was assessed with 5 items (area A—Health and safety at work and work-related stress), an example is “My workplace is safe and comfortable”. Cronbach’s alpha for the scale in this study was 0.75 for clinical staff and 0.75 for non-clinical staff.

Sense of belonging was assessed with 4 items (area H—The sense of belonging), an example is “I am proud to work in this organization”; Cronbach’s alpha was 0.87 for clinical staff and 0.83 for non-clinical staff.

Lack of discrimination was assessed with 7 items (area B—Discrimination), an example is “I am treated correctly and with respect in relation to my political orientation”; Cronbach’s alpha was 0.91 for clinical staff and 0.92 for non-clinical staff.

Fairness was assessed with 5 items (area C—Fairness in my administration), an example is “I believe there is fairness in assigning work to do”; Cronbach’s alpha was 0.86 for clinical staff and 0.88 for non-clinical staff.

Career and professional development was assessed with 6 items (area D—Career and professional development), an example is “In my organization, the path of workers’ professional development is well defined and clear”; Cronbach’s alpha was 0.87 for clinical staff and 0.88 for non-clinical staff.

Job autonomy was assessed with 5 items (area E—My work), an example is “I have an adequate level of autonomy in carrying out my work”; Cronbach’s alpha was 0.75 for clinical staff and 0.78 for non-clinical staff.

Organizational goals’ sharing was assessed with 4 items (area L—My organization), an example is “I agree with the strategic goal of my administration”; Cronbach’s alpha was 0.89 for clinical staff and 0.90 for non-clinical staff.

The questionnaire also collected participants’ demographic data: Gender, age, organizational tenure, qualifications, and job.

### 2.3. Data Analysis

Descriptive analysis was carried out in each sample separately, using the statistics software, IBM SPSS 24 (IBM, Armonk, NY, USA). Pearson correlations were used to examine the interrelationships between variables. Cronbach’s alpha coefficient was calculated to test the reliability of each scale. Differences in the means of variables between the two groups were examined using the analysis of variance (*t*-test for independent samples). Psychometric properties of the scales were evaluated through a confirmatory factor analysis (maximum likelihood method of estimation) using Mplus 7 (Muthén & Muthén, Los Angeles, CA, USA) [34].

The multi-group structural equation model (SEM) was performed using Mplus 7 [34] to assess differences across both samples in the hypothesized model. The method of estimation was maximum likelihood (ML). According to the literature [35], the model was assessed using several goodness-of-fit criteria: The *χ*^2^ goodness-of-fit statistic; the root mean square error of approximation (RMSEA); the comparative fit index (CFI); the Tucker Lewis index (TLI); and the standardized root mean square residual (SRMR). Non-significant values of *χ*^2^ indicate that the hypothesized model fits the data. Values of RMSEA smaller than 0.05 indicate a good fit, values smaller than 0.08 indicate an acceptable fit, and values greater than 1 should lead to model rejection. CFI and TLI values greater than 0.95 indicate a good fit. The SRMR ranges from 0 to 1, with a cut-off criterion of 0.08, with higher values indicating an inferior fit to the empirical data and values lower than 0.05 indicating an excellent fit. 

To address the common method variance issue, we performed the Harman’s single-factor test [36] using confirmatory factor analysis. Results indicate that one single factor could not account for the variance in the data, since all measures of goodness-of-fit show that the model did not fit the data (*χ^2^*(665) = 11,892.97, *p* < 0.01, RMSEA = 0.12 (90% confidence interval (CI) 0.11, 0.12), CFI = 0.53, TLI = 0.50, SRMR = 0.11); therefore, the threat of common method bias is unlikely.

## 3. Results

Table 2 shows means, standard deviations, and correlations among the variables, and the internal consistency of each scale for the clinical staff sample, and Table 3 shows the same data for the non-clinical staff sample.

All *α* values meet the criterion of 0.70, ranging between 0.75 and 0.92. Some variables are highly correlated: We conducted multicollinearity analysis using the variance inflation factors (VIFs), which should be less than 10 [37,38] All the independent variables had VIFs < 10, indicating that multicollinearity is not an issue.

All the significant correlations between the variables were in the expected directions. Health and safety at work in both samples was positively associated with lack of discrimination (clinical: *r* = 0.33, *p* < 0.01; non-clinical: *r* = 0.35, *p* < 0.01), fairness (clinical: *r* = 0.51, *p* < 0.01; non-clinical: *r* = 0.51, *p* < 0.01), career and professional development (clinical: *r* = 0.50, *p* < 0.01; non-clinical: *r* = 0.44, *p* < 0.01), job autonomy (clinical: *r* = 0.45, *p* < 0.01; non-clinical: *r* = 0.43, *p* < 0.01), and organizational goals’ sharing (clinical: *r* = 0.38, *p* < 0.01; non-clinical: *r* = 0.39, *p* < 0.01). 

Sense of belonging in both samples was positively associated with a lack of discrimination (clinical: *r* = 0.31, *p* < 0.01; non-clinical: *r* = 0.29, *p* < 0.01), fairness (clinical: *r* = 0.51, *p* < 0.01; non-clinical: *r* = 0.49, *p* < 0.01), career and professional development (clinical: *r* = 0.59, *p* < 0.01; non-clinical: *r* = 0.53, *p* < 0.01), job autonomy (clinical: *r* = 0.57, *p* < 0.01; non-clinical: *r* = 0.47, *p* < 0.01), and organizational goals’ sharing (clinical: *r* = 0.51, *p* < 0.01; non-clinical: *r* = 0.54, *p* < 0.01).

Health and safety at work and sense of belonging were positively correlated in both groups (clinical: *r* = 0.43, *p* < 0.01; non-clinical: *r* = 0.24, *p* < 0.01).

Analysis of variance between the two samples showed differences in career and professional development and job autonomy: Individuals working in clinical staff perceived more career and professional development (*M* = 3.11, *SD* = 1.28) than individuals working in non-clinical staff (*M* = 2.77, *SD* = 1.28) [*t* (1154) = 3.62, *p* < 0.001]. Moreover, clinical staff perceived more job autonomy (*M* = 4.59, *SD* = 0.93) than non-clinical staff (*M* = 4.19, *SD* = 1.08) [*t* (1154) = 5.51, *p* < 0.001]. In clinical and non-clinical samples, some workers had a supervisory role. Analysis of variance was conducted to verify any differences based on the supervisory role. No statistically significant differences emerged within the two groups considered.

**Figure 2:**
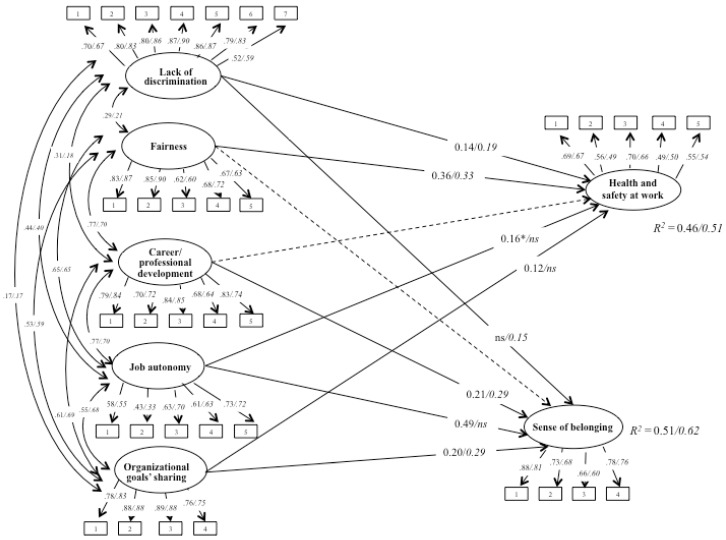
The structural equation model, standardized path coefficients (*p* < 0.01, * *p* < 0.05). Results of the multi-group analysis: Clinical staff/non-clinical staff. Discontinuous lines indicate a non-significant relationship.

The multi-group SEM of the hypothesized model (Figure 1) fit the data well: *χ*² (1126, N_clinical_ = 939, N_non-clinical_= 231) = 3035.70, *p* = 0.001, CFI = 0.92, TLI = 0.92, RMSEA = 0.05 (90% CI 0.05, 0.06), SRMR = 0.06. Figure 2 shows the standardized parameters. By examining the estimated model, the variables showed good item loadings in both groups. 

Hypothesis 1 stated that job resources are related to health and safety at work: Results confirmed the relation for a lack of discrimination and fairness in both groups, and for job autonomy and organizational goals’ sharing only in clinical staff. In detail, a lack of discrimination showed a significantly positive relationship with health and safety at work in both groups (clinical: *β* = 0.14, *p* < 0.01; non-clinical: *β* = 0.19, *p* < 0.01). Fairness showed a strong significantly positive relationship with health and safety at work in both groups (clinical: *β* = 0.36, *p* < 0.01; non-clinical: *β* = 0.33, *p* < 0.01). Career and professional development did not show a significant relationship with health and safety at work in either sample. Job autonomy showed a significant relationship with health and safety at work only among the clinical staff (*β* = 0.16, *p* < 0.05). Organizational goals’ sharing also showed a significant relationship with health and safety at work only among the clinical staff (*β* = 0.12, *p* < 0.01). 

Regarding sense of belonging, hypothesis 2 stated that job resources are related to it: Results confirmed the relation for career and professional development and organizational goals’ sharing in both groups, and for lack of discrimination only in non-clinical staff and job autonomy only in clinical staff. Lack of discrimination showed a significant positive relationship with sense of belonging only in the non-clinical group (*β* = 0.15, *p* < 0.01). Fairness did not show a significant relationship with sense of belonging in either sample. Career and professional development showed a significantly positive relationship with sense of belonging in both groups (clinical: *β* = 0.21, *p* < 0.01; non-clinical: *β* = 0.29, *p* < 0.01). Job autonomy showed a strong significant relationship with sense of belonging only in the clinical group (*β* = 0.49, *p* < 0.01). Organizational goals’ sharing showed a significant relationship with sense of belonging in both groups (clinical: *β* = 0.20, *p* < 0.01; non-clinical: *β* = 0.29, *p* < 0.01)).

The variance of the dependent variables explained by the models was 46% for health and safety at work, and 66% for sense of belonging in the clinical staff sample, and 51% for health and safety at work, and 62% for sense of belonging in the non-clinical staff sample.

## 4. Discussion

This study was designed to assess well-being at work in an Italian hospital, according to a legislative directive on this topic (Legislative Decree 150/2009). Moreover, the study aims at improving the assessment of well-being at work, starting from the ANAC questionnaire and recommendations [12]. The tool proposed by ANAC is widely used by many Italian public agencies, but few studies have analyzed its theoretical framework or actions taken as a result of these assessments [11]. This study attempts to improve and expand this issue. 

This study involved an Italian teaching hospital, a specific health care context. In health care services, organizational success and performance are achieved through a combination of several factors related to human, relational, and structural aspects of the organization. Professionals with their knowledge, skills, and abilities are employed by the institution to deliver quality care to patients, and the overall organization’s performance depends on the performance and well-being of its employees [39]. Health care staff is also considered as a professional group with a high risk of work-related stress and job dissatisfaction [20,40]. Several studies showed that high levels of stress at work are connected to higher absenteeism rates and decreased performance in service workers [2]. Moreover, many studies have analyzed factors that can enhance well-being, motivation, and job satisfaction [6,13]: Frequently, they are connected to organizational and relational factors, such as a clear organizational vision and mission, good relationships with supervisors, a well-organized job design, and adequate wages and working conditions [41]. Moreover, there is increasing evidence to support the importance of employee engagement in enhancing organizational performance in the health care sector [8,18,19,42].

According to the results, the correlations and multi-group structural equation model showed that health and safety at work and sense of belonging had significant positive relationships with the job resources considered. Moreover, some differences emerged between the determinants of the two outcomes and among the two groups. Health and safety at work showed a significant positive relationship with several job resources: Fairness and lack of discrimination in both groups, and job autonomy and organizational goals’ sharing only in the clinical group. Sense of belonging presented a significant positive relationship with job resources: Career and professional development and organizational goals’ sharing in both groups, and lack of discrimination only in the non-clinical group and job autonomy only in clinical group. 

The results also underlined the importance of the role of all the variables considered in this study in relation to the well-being outcome. Some differences emerged, but all the job resources had significant positive relationships with health and safety at work and/or sense of belonging. Lack of discrimination appeared as a job resource that could sustain well-being at work and reinforce the sense of belonging [21]. Fairness showed a strong relationship only with health and safety at work. Several studies found that this resource is related to citizenship behavior and commitment [24,25]. Future research could expand upon these findings. Career and professional development showed a strong relationship with sense of belonging, according to previous studies that investigated the role of this job resource in relation to well-being and work-related stress [27]. Job autonomy was strongly related to both outcomes: The results suggested guaranteeing employees’ freedom and independence in scheduling work and activities [29,30]. Likewise, organizational goals’ sharing was related to both outcomes. The results underlined the importance for organizations to share their goals, mission, and values [31]. In fact, a systematic information sharing process provided by top management (regarding, for example, goals, performance, and organizational structure) seems to be an effective strategy to engage employees [43] and promote well-being and health in the workplace. Results underlined the important role of job resources that are mainly responsible for motivational processes, within the JD-R theory [6].

Our results allowed reflections about the two groups considered, clinical staff and non-clinical staff. In the clinical staff group, for example, important role of job autonomy emerged, which seems to be able to enhance health and safety at work and sense of belonging. For non-clinical staff, however, job autonomy did not have a significant relationship with the outcomes, and this resource seems to play a less central role for the workers’ well-being. Moreover, lack of discrimination had a significant role with sense of belonging only in the non-clinical staff; it appears to be not significant and central for the well-being in the other group. Future research, also qualitative, could investigate the potential differences between the different groups in the organization, considering also the specific and typical job demands of clinical and non-clinical staff workers.

### Limitations and Future Research

Among the limitations of the study, the use of a cross-sectional research design did not permit causal inferences to be made on the relationships between variables, but only to hypothesize the direction. Furthermore, using self-report measures may have inflated correlations between variables [36]. We performed Harman’s single-factor test to control this aspect and the threat of common method bias was unlikely. 

The research was conducted in a single organization. In future research, it would be useful to test the same relationships in other organizations, in both the public and private sectors.

Furthermore, in this study, we only considered job resources because the ANAC tool did not comprise job demands. In future studies, it would be important to use both qualitative and quantitative research tools that take into account not only resources, but also job demands [6] and the psychosocial risks defined as “emerging” [44]. Many studies have analyzed them in relation to well-being and work-related stress, such as the safety climate [15], job insecurity [45], emotional demands of work [32], work-life balance, and the use of new technologies [46,47]. Moreover, it would be important to consider several risks and demands typical of the health care sector, for example, the workload, patient care and efficiency, and also demographic characteristics, for example, those related to aging [48].

A further limitation concerns the research tool, the ANAC questionnaire, for the assessment of organizational well-being. As stated, there is no indication or measurement for psychometric and reliability properties from the National Anti-Corruption Authority that proposed the ANAC questionnaire. Moreover, only a few studies have used this tool [11]. In this study, we achieved good results in the measurement model, factors loadings, and reliability of variables considered, but it would be necessary to deepen the psychometric characteristics of the ANAC tool for the assessment of well-being at work. Future research should evaluate the reliability of the tool as a whole, also in other work contexts or professional sectors.

## 5. Conclusions

Starting from the results presented and discussed above, it is possible to identify some reflections and indications regarding the assessment of well-being in the health care sector and potential implications for theory and practice. Mainly, the results showed the importance to design interventions to prevent work-related stress and to improve health, well-being, and performance of health care employees [40,49].

First, results highlighted the importance of managing and monitoring the evaluation of work-related stress and well-being. It appears significant to try to integrate regulatory indications and research practice: The implementation of interventions in organizational systems can promote employees’ well-being and sustain economic performance of the organization as a whole [3,8,14]. Our results suggested the importance of the supervision and maintenance of health and safety in the workplace; organizations will benefit from risk assessments in order to find ways to mitigate or avoid the risks and hazards inherent in their particular environments.

The study emphasized the fundamental role of various job resources in relation to well-being at work, for both the job categories considered. In particular, organizations should monitor and make accessible the resources to support motivational processes that can foster well-being and satisfaction [6,8,10]. The results underlined the importance of ensuring adequate job autonomy [28,50], in particular for clinical staff, as emerged in several studies that highlighted the strong relationship among the degree of autonomy, job satisfaction, and quality of care [51,52].

Results suggested that attention should be paid to the perception of fairness, career opportunities, and professional development, which could be supported and monitored through specific projects or initiatives aimed at different categories of workers. Referring to career opportunities and professional development, for example, clarity and information sharing may compensate for the potential negative effect of other risk factors or job demands, such as workload or role ambiguity [27]. In organizations, the monitoring of information transfer and sharing is identified as being important, and career support for professional development should be offered. This can be particularly useful to sustain a sense of belonging in both groups considered.

Moreover, management could promote training to foster a supportive work environment for employees [4,5,49,53]. Organizations can develop this supportive environment by training supervisors to be better leaders, emphasizing the importance of fairness, teamwork, and social support, and establishing the value of safety.

Finally, it is important to underline the fundamental role of training that at all levels and for all groups of employees, could improve diversity management and guarantee an “inclusive workplace” [22]: The results identify the relationship between a lack of discrimination and health and safety at work and a sense of belonging. Regarding the issue of discrimination, organizations could activate specific company policies, in addition to ethical codes, to create work environments that are attentive to workers’ well-being and are inclusive for all employees [21,54].

## Figures and Tables

**Table 1:** Confirmatory factor analysis results.

Measures	CFA Results	Alpha
Health and safety at work	χ^2^(3) = 26.49, *p* < 0.05; RMSEA = 0.08; CFI = 0.99; TLI = 0.99; SRMR = 0.02	0.75
Sense of belonging	χ^2^(2) = 21.48, *p* < 0.01; RMSEA = 0.08; CFI = 0.99; TLI = 0.95; SRMR = 0.01	0.86
Lack of discrimination	χ^2^(13) = 62.00, *p* < 0.01; RMSEA = 0.06; CFI = 0.99; TLI = 0.98; SRMR = 0.02	0.92
Fairness	χ^2^(4) = 13.12, *p* = ns; RMSEA = 0.04; CFI = 0.99; TLI = 0.99; SRMR = 0.01	0.86
Career/professional development	χ^2^(4) = 18.53, *p* < 0.01; RMSEA = 0.06; CFI = 0.99; TLI = 0.99; SRMR = 0.01	0.88
Job autonomy	χ^2^(4) = 15.98, *p* < 0.05; RMSEA = 0.05; CFI = 0.99; TLI = 0.98; SRMR = 0.02	0.76
Organizational goals’ sharing	χ^2^(2) = 6.69, *p* < 0.05; RMSEA = 0.04; CFI = 0.99; TLI = 0.99; SRMR = 0.01	0.89

Notes: *N* = 1170; CFA = Confirmatory Factor Analysis; ns = not significant; all standardized loadings ranged from 0.52 to 0.89.

**Table 2:** Means, standard deviations, and correlations for the clinical staff sample.

Variables	Clinical	Correlations						
	M (SD)	1	2	3	4	5	6	7
1. Health and safety at work	3.70 (1.09)	*0.75*						
2. Sense of belonging	4.20 (1.24)	0.43 **	*0.87*					
3. Lack of discrimination	5.40 (0.95)	0.33 **	0.31 **	*0.91*				
4. Fairness	2.85 (1.27)	0.51 **	0.51 **	0.28 **	*0.86*			
5. Career/professional development	3.11 (1.28)	0.50 **	0.59 **	0.32 **	0.71 **	*0.87*		
6. Job autonomy	4.59 (0.93)	0.45 **	0.57 **	0.40 **	0.50 **	0.60 **	*0.75*	
7. Organizational goals’ sharing	2.75 (1.31)	0.38 **	0.51 **	0.17 **	0.48 **	0.53 **	0.43 **	*0.89*

Notes: M = means, SD = standard deviations; Cronbach’s α for clinical staff sample are on the diagonal. ** *p* < 0.01.

**Table 3:** Means, standard deviations, and correlations for the non-clinical staff sample.

Variables	Non-Clinical	Correlations						
	M (SD)	1	2	3	4	5	6	7
1. Health and safety at work	3.83 (1.09)	*0.75*						
2. Sense of belonging	4.18 (1.31)	0.24 **	*0.83*					
3. Lack of discrimination	5.28 (1.14)	0.35 **	0.29 **	*0.92*				
4. Fairness	2.99 (1.34)	0.51 **	0.49 **	0.22 **	*0.88*			
5. Career/professional development	2.77 (1.28)	0.44 **	0.53 **	0.17 *	0.64 **	*0.88*		
6. Job autonomy	4.19 (1.08)	0.43 **	0.47 **	0.38 **	0.47 **	0.54 **	*0.78*	
7. Organizational goals’ sharing	2.83 (1.38)	0.39 **	0.54 **	0.16 *	0.54 **	0.59 **	0.55 **	*0.90*

Notes: M = means, SD = standard deviations; Cronbach’s α for non-clinical staff sample are on the diagonal. * *p* < 0.05; ** *p* < 0.01.
